# Curdlan Prevents the Cognitive Deficits Induced by a High-Fat Diet in Mice via the Gut-Brain Axis

**DOI:** 10.3389/fnins.2020.00384

**Published:** 2020-05-14

**Authors:** Xiaoying Yang, Mingxuan Zheng, Shanshan Hao, Hongli Shi, Danhong Lin, Xi Chen, Alec Becvarovski, Wei Pan, Peng Zhang, Minmin Hu, Xu-Feng Huang, Kuiyang Zheng, Yinghua Yu

**Affiliations:** ^1^Jiangsu Key Laboratory of Immunity and Metabolism, Department of Pathogen Biology and Immunology, Xuzhou Medical University, Xuzhou, China; ^2^Illawarra Health and Medical Research Institute (IHMRI), School of Medicine, University of Wollongong, Wollongong, NSW, Australia

**Keywords:** curdlan, dietary fiber, cognition, gut-brain axis, high-fat diet, obesity

## Abstract

A high-fat (HF) diet is a major predisposing factor of neuroinflammation and cognitive deficits. Recently, changes in the gut microbiota have been associated with neuroinflammation and cognitive impairment, through the gut-brain axis. Curdlan, a bacterial polysaccharide widely used as food additive, has the potential to alter the composition of the microbiota and improve the gut-brain axis. However, the effects of curdlan against HF diet-induced neuroinflammation and cognitive decline have not been investigated. We aimed to evaluate the neuroprotective effect and mechanism of dietary curdlan supplementation against the obesity-associated cognitive decline observed in mice fed a HF diet. C57Bl/6J male mice were fed with either a control, HF, or HF with curdlan supplementation diets for 7 days (acute) or 15 weeks (chronic). We found that acute curdlan supplementation prevented the gut microbial composition shift induced by HF diet. Chronic curdlan supplementation prevented cognitive declines induced by HF diet. In addition, curdlan protected against the HF diet-induced abnormities in colonic permeability, hyperendotoxemia, and colonic inflammation. Furthermore, in the prefrontal cortex (PFC) and hippocampus, curdlan mitigated microgliosis, neuroinflammation, and synaptic impairments induced by a HF diet. Thus, curdlan—as a food additive and prebiotic—can prevent cognitive deficits induced by HF diet via the colon-brain axis.

## Introduction

Obesity, a major global health concern, is not only closely associated with type 2 diabetes mellitus, cardiovascular disease, cancer, depression, and other chronic diseases but is also a risk factor for neurodegenerative diseases, such as Alzheimer’s disease (AD) and vascular dementia (Fruh, 2017; O’Brien et al., 2017). Neuroinflammation is a hallmark of neurodegenerative diseases associated with high-fat (HF) diet (Francis and Stevenson, 2013; Subhramanyam et al., 2019). Gut microbiota play an important role in brain function and behaviors through the gut-brain axis, while a dysbiosis of the microbiota is crucially involved in neuroinflammation and cognitive impairment (Erny et al., 2015; Sharon et al., 2016). For example, brain development is abnormal when the gut microbiota is absent in germ-free animals (Gareau et al., 2011; Clarke et al., 2013). Previous research in germ-free and antibiotic-treated, pathogen-free rodents has shown that gut microbiota dysbiosis negatively influences hippocampal neurogenesis and brain development through the activation of microglia (Erny et al., 2015; Sharon et al., 2016). Therefore, a dysbiosis of the gut microbiota is evidently a key initiating factor of neuroinflammation and subsequent neuronal dysfunction (Frohlich et al., 2016; Rogers et al., 2016; Sharon et al., 2016).

The gut microbiota serves as an important regulator for host intestinal homeostasis and immunity. For example, *Bacteroides fragilis* of Bacteriodetes phylum increases tight junction proteins expression and attenuates intestinal permeability (Hsiao et al., 2013), while *Ruminococcus* of Firmicutes phylum degrades mucus (Hynonen et al., 2016). Intestinal barrier dysfunction is marked by an increase in permeability, which allows the translocation of bacteria or bacterial lipopolysaccharide (LPS, endotoxin) into the blood circulation, which may stimulate an immune response resulting in positive feedback inflammation and tissue damage in the intestine (Armstrong et al., 2018; Zhang et al., 2019). Furthermore, it is reported that the intraperitoneal injection of LPS activates microglia and induces elevation of pro-inflammatory cytokines in the brains of mice (Chen et al., 2018). Chronic HF diet-induced obesity in mice has been associated with gut microbiota dysbiosis, impaired intestinal barrier integrity, and elevated plasma lipopolysaccharide (Serino et al., 2012; Zhang et al., 2019). Additionally, the hyperendotoxemia observed with intestinal disorders could trigger neuroinflammation and lead to cognitive impairment (Lee et al., 2008; Chen et al., 2018). Therefore, the dysregulation of the gut-brain axis is considered as the potential mechanism by which a chronic HF diet induces neuroinflammation and cognitive impairment (Wang et al., 2017; Zhang et al., 2019). In clinical studies, intestinal alterations, hyperendotoxemia, and neuroinflammation have been shown in obese individuals, AD patients, and individuals with brain amyloidosis (Cattaneo et al., 2017; Cryan and Dinan, 2012; Ley et al., 2006). Meanwhile, dietary supplementation with probiotics or prebiotics can prevent cognitive impairment via the microbiota-gut-brain axis (Wang et al., 2016; Jiang et al., 2017; Li et al., 2018; Brett and de Weerth, 2019; Sun et al., 2019). Therefore, this axis is a feasible target for the prevention and treatment of cognitive impairment induced by HF diet.

Curdlan is one of the few bacterial additives approved by the US Food and Drug Administration (FDA), which is produced by only bacteria belonging to the *Agrobacterium* and *Alcaligenes* species (Shih et al., 2009). Curdlan becomes “curdle” when heated; therefore, such a property enables it to be used as a gelling material to improve the textural quality, water-holding capacity, and thermal stability of various foods. Currently, curdlan is widely used as an additive for noodles, sauces, frozen foods, and packaged meats (Spicer et al., 1999; Mangolim et al., 2017). Additionally, curdlan has growing potential in the pharmaceutical industry because of its potent biological activities. Curdlan inhibits malarial merozoite invasion and is considered a potential auxiliary treatment for severe malaria (Evans et al., 1998); It has been found to exhibit high antiviral (HIV and Dengue Virus) activity with low side effects (Jagodzinski et al., 1994; Ichiyama et al., 2013). Curdlan has also been found to trigger neuronal axon regeneration and have neuroprotective effects (Baldwin et al., 2015). Curdlan is an insoluble polysaccharide composed of linear β-(1,3)-glucan. Polysaccharides are unable to be processed by gut enzymes of the hosts, but can be fermented by specific intestinal microbiota (Zhang et al., 2018). Thus, polysaccharides serve as unique carbon sources for specific gut microbiota during fermentation. Furthermore, degradation of polysaccharides produces a large number of oligosaccharides that are conducive to host gut health (Zhang et al., 2018). Therefore, as a polysaccharide, curdlan also has the potential to regulate gut microbiota and gut-brain axis. However, it remains unknown whether dietary curdlan can ameliorate the cognitive impairments induced by HF diet, via gut-brain axis.

In the present study, a HF diet mouse model was employed to induce cognitive impairments and subsequently evaluate the effects of dietary curdlan supplementation on various parameters including composition of the gut microbiota, colonic mucus thickness and tight junction protein, and serum endotoxin (LPS) level. Additionally, the pro-cognitive efficacy of curdlan in this model was evaluated by examining neuroinflammation, synaptic protein levels, and ultrastructure of the prefrontal cortex (PFC) and hippocampus, which are two key areas involved in cognition. The present study provides first evidence that dietary curdlan supplementation can ameliorate gut dysbiosis and protect against the cognitive impairment in diet-induced obesity, via the gut-brain axis.

## Materials and Methods

### Animals and Treatment

Sixty male C57BL/6J mice aged 9 weeks were purchased from the Experimental Animal Center of Xuzhou Medical University [Xuzhou, China, SCXK (Su) 2015-0009], and were housed and maintained in a 12 h light/dark photoperiod with unrestricted access to water and food. After habituation to the laboratory environment for 1 week, the mice were randomly divided into two experiments: acute experiment and chronic experiment (*N* = 30 per experiment). For each experiment, mice were further divided into three groups (*N* = 10 per group): (1) mice fed a lab chow diet [low-fat (LF) diet, 5% fat by weight] as a control (LF) group; (2) mice receiving the HF diet (30% fat by weight) as the HF group; and (3) mice fed the HF diet supplemented with curdlan from *Alcaligenes faecailis* (500 mg/kg food, Sigma-Aldrich, St. Louis, MO, United States) (Qi et al., 2011) as the HFCurd group. For acute experiments, mice received the respective diets for 7 days. Body weight and food intake were recorded every day. Mice were then euthanized, their cecal contents collected and stored at −80°C for further analyses. For chronic experiments, mice were administered the three diets for 15 weeks. Body weight and food intake were measured on the last day of each week. The cognitive behavior tests were performed (*N* = 10 per group), including the temporal order memory test at 13th week of feeding, the novel object recognition test at 14th week, and the Y-maze test at 15th week. Mice were sacrificed 4 days after behavioral testing with CO_2_. The experimental timeline is shown in Supplementary Figure 1. Liver and fat pads (subcutaneous, epididymal, and brown) were dissected and weighed. Blood serum, intestinal, liver, and brain tissues were also collected and stored in −80°C for further analyses. All animal care and experiments were carried out under protocols approved by the ethics committee of Xuzhou Medical University.

### Gut Microbiota Analysis

DNA was extracted from the cecal contents and 16S rRNA sequencing was conducted as previously described (Sun et al., 2019). The genomic DNA in the cecal contents was extracted by using the HiPure stool DNA kit (Magen, Beijing, China). The V4 region of the 16S rRNA genes were amplified and sequenced on the Illumina Miseq 2500 platform (Shanghai Majorbio Biopharm Technology Co., Ltd., Shanghai, China). The operational taxonomic units (OTUs) and the taxonomy of each 16S rRNA gene sequence were analyzed as previously reported (Zhang et al., 2019).

### LPS Determination

Serum concentration of LPS was determined using a chromogenic end-point TAL kit (Xiamen Bioendo Technology Co., Ltd., Xiamen, China). The absorbance was measured at 545 nm using a spectrophotometer (Asuragen ClinBio128, United States), with measurable concentrations ranging from 0.1 to 1.0 EU/ml. All samples for LPS measurements were performed in duplicate.

### Histological Staining and Immunohistochemistry

For detection of colonic mucus layer thickness, colonic tissues were fixed in Carnoy’s solution and stored in methanol, before dehydration and embedding in paraffin. After fixation, the colon was cut in 5 μm sections with Alcian blue staining as previously described (Desai et al., 2016). The thickness of the colonic mucus layer was measured using ImageJ software. The immunohistochemical staining has been described in our previous study (Zhang et al., 2019). Briefly, fixed colon tissues were embedded in paraffin and sectioned at 5 μm. The sections were rehydrated in xylene and then in graded ethanol solutions. The sections were then washed in 3% H_2_O_2_ in methanol for 30 min. For brain tissues, fixed tissues were sectioned at 20 μm, washed three times with phosphate bufer saline (PBS) for 10 min, and then washed in 1% H_2_O_2_ in PBS for 30 min. All sections were blocked with 5% normal goat serum and incubated with indicated primary antibodies at 4°C overnight. Primary antibodies were anti-F4/80 (ab16911, abcam, United Kingdom, 1: 1,000 dilution) for the colon and anti-Iba1 (019-19741, Wako Pure Chemical Industries, Japan, 1: 1,000 dilution) for the brain. Sections were washed with PBS and then incubated with goat anti-rabbit IgG H&L (ab6702, abcam, United Kingdom, 1: 500 dilution) for 2 h at room temperature. Finally, the sections were washed and developed using the DAB peroxidase substrate kit (Cell Signaling Technology, Boston, MA, United States), and counterstained with hematoxylin (Sigma-Aldrich, St. Louis, MO, United States). All sections were imaged under a microscope (OLYMPUS IX51, Tokyo, Japan), and digital photographs were captured. ImageJ software was used to quantify the area of F4/80 or Iba1 immunoreactivity on each field.

### Behavioral Testing

The temporal order memory, novel object recognition, and Y-maze tests were performed to examine dietary effects on spatial and recognition memory based on methods previously described (Jin et al., 2016; Uribe-Marino et al., 2016; Zhang et al., 2019). In the temporal order memory test, the discrimination index was calculated as [(Time with the older object – Time with recent object)/Total time with both objects] × 100. In the novel object recognition test, the discrimination index was evaluated by using the formula, [(Time with recent object - Time with the older object)/Total time with both objects] × 100. For the Y-maze test, the alternation was defined as the successful successive entry into each of the three arms. The alternation triplet (%) was calculated as [number of successful alternations/(total number of arms entries –2) × 100].

### Transmission Electron Microscopy (TEM)

After transcardial perfusion with saline, brain tissues were taken out and 1 mm^3^ of tissue blocks from PFC and the CA1 regions of hippocampus were dissected. Samples were fixed in a 2% paraformaldehyde-2.5% glutaraldehyde mixture for 24 h and treated post-fixation with 1% osmium tetroxide (OsO4) for 2 h, before dehydration in an ascending graded ethanol series and embedding in epoxy resin. Sections (70 nm) were cut and stained with 4% uranyl acetate and 0.5% lead citrate. Ultrastructure of synapses in the PFC and CA1 region of the hippocampus was measured under a transmission electron microscope (FEI, Portland, OR, United States), and synaptic morphometrics were studied. Postsynaptic density, synaptic clefts width, and the curvature of the synaptic interface were determined using ImageJ software as described previously (Wu et al., 2013).

### Western Blotting

Western blot assays were performed as described previously (Xu et al., 2018). Briefly, proteins were extracted from colon, hippocampus, and PFC in cell lysis buffer containing RIPA buffer (Sigma-Aldrich, St. Louis, MO, United States), Protease Inhibitor Cocktail (Sigma-Aldrich, St. Louis, MO, United States), and 1 mM PMSF (Sigma-Aldrich, St. Louis, MO, United States). After quantification by BCA assay (Beyotime Biotech, Beijing, China), 40–80 μg of protein was separated using 10% SDS-PAGE and then electrotransferred to polyvinylidene difluoride (PVDF) membrane (BioRad, Hercules, CA, United States). Western blot assays were performed using primary antibodies specific for Occludin (ab167161, abcam, United Kingdom, 1: 2,000 dilution), brain derived neurotrophic factor (BDNF) (ANT-010, Alomone labs, Israel, 1:200 dilution), PSD-95 (#3450, Cell Signaling Technology, Boston, MA, United States, 1: 1,000 dilution), and GAPDH (A2077, ABclonal Biotechnology Co., Ltd., United States, 1:2,000 dilution). Secondary antibody was anti-rabbit IgG conjugated with horseradish peroxidase (sc-2030, Santa Cruz Biotechnology, United States, 1:2,000 dilution). Immunodetection was performed using Clarity^TM^ ECL western blot substrate (Bio-Rad, United States) and visualized with the ChemiDoc Touch imaging system (Bio-Rad, United States).

### RNA Extraction and Quantitative (q) Real-Time PCR (qPCR)

RNA extraction and qPCR were performed based on methods previously described (Mei et al., 2019). Briefly, total RNA was extracted with TRIzol (Thermo Fisher Scientific, United States) from the colon, hippocampus, and PFC. Then, 2 μg RNA was reverse-transcripted to cDNA using a high-capacity cDNA reverse transcription kit (Takara, Japan). qPCR was performed using the SYBR GREEN Master Mix (TaKaRa, Japan) and determined on a real-time PCR detection system (Bio-Rad, United States). The mRNA levels for specific genes were calculated using the formula 2^(–ΔΔCt)^ and normalized by β-actin mRNA levels. All primers are listed in Supplementary Table 1.

### Statistical Analysis

The data are presented as means ± SEM. Statistical analysis was performed using the one-way analysis of variance (ANOVA) by SPSS (version 20, IBM Corporation, Chicago, IL, United States), followed by the *post hoc* Tukey test for comparisons among the groups. A *p* value<0.05 was considered as statistically significant.

## Results

### Acute Curdlan Supplementation Attenuated the Gut Microbiota Dysbiosis Induced by HF Diet

**FIGURE 1:**
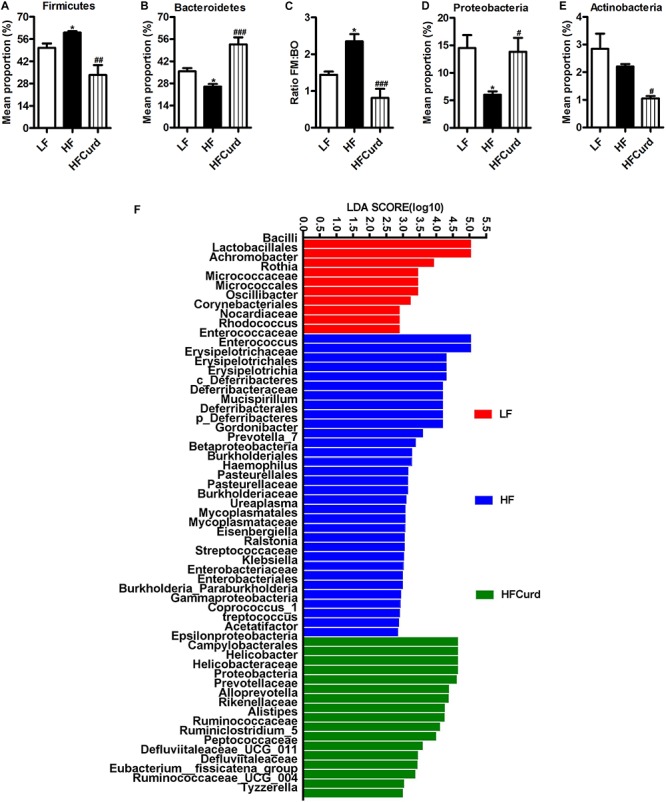
Effects of acute curdlan supplementation on energy intake, body weight, and gut microbial communities in mice fed a high-fat (HF) diet for 7 days. **(A)** Relative abundance of Firmicutes, **(B)** relative abundance of Bacteroidetes, **(C)** ratio of Firmicutes proportion to Bacteroidetes proportion (Ratio FM/BO), **(D)** relative abundance of Proteobacteria, and **(E)** relative abundance of Antinobacteria. Values are mean ± standard error of means. *n* = 5. *p < 0.05 vs. LF. ^#^*p* < 0.05 vs. HF. ^##^*p* < 0.01 vs. HF. ^###^*p* < 0.001 vs. HF. **(F)** With linear discriminant analysis (LDA) combined with effect size measurements (LEfSe), 59 distinctively affluent taxonomic classes of microbiota were revealed to enable discrimination among three groups. The LDA score >2.0 was considered significant.

Studies in human and rodents have shown that chronic HF diet induced microbial dysbiosis with regards to diversity and composition (Lynch and Pedersen, 2016; Zhang et al., 2019). Here, we examined the gut microbiota after an acute HF diet with or without curdlan supplementation for 7 days by 16S rRNA sequencing. After acute dietary intervention, there is no significant difference observed in the richness (represented by the Chao index) and diversity (represented by the Shannon index) of gut microbiota among the three groups: control, HF, and curdlan supplementation groups [*F*_(__2_,_12__)_ = 2.769, *p* = 0.1026, Supplementary Figure 2A; *F*_(__2_,_12__)_ = 0.9687, *p* = 0.4074, Supplementary Figure 2B]. However, curdlan prevented the microbial composition shift induced by HF diet (Figures 1A–F). At the phyla levels, the HF diet group’s microbiota at 7 days were characterized by elevated abundance of Firmicutes, decreased abundance of Bacteroidetes, increased ratio of Firmicutes to Bacteroidetes, and decreased abundance of Proteobacteria [*F*_(__2_,_12__)_ = 5.649, *p* = 0.0187, Figure 1D]; while curdlan ameliorated these abnormities in microbial composition induced by HF diet [*F*_(__2_,_12__)_ = 8.273, *p* = 0.0055, Figure 1A; *F*_(__2_,_12__)_ = 29.22, *p* < 0.001, Figure 1B; *F*_(__2_,_12__)_ = 14.37, *p* = 0.0007, Figure 1C; *F*_(__2_,_12__)_ = 5.649, *p* = 0.0187, Figure 1D]. The abundance of Actinobacteria was also significantly decreased by curdlan [*F*_(__2_,_12__)_ = 13.63, *p* = 0.0011, Figure 1E].

Using LDA effect size (LEfSe) calculation to compare the microbiota of three groups, we observed 59 distinctively affluent taxonomic classes with an LDA score higher than 2.0 (Figure 1F). Notably, bacteria belonging to family *Prevotellaceae*, genus *Alloprevotella*, family *Rikenellaceae*, and genus *Alistipes* (the lower taxa of phylum Bacteroidetes) were elevated significantly in HFCurd mice. Furthermore, phylum Proteobacteria, class Epsilonproteobacteria, order Campylobacterales, family *Helicobacteraceae*, family *Ruminococcaceae*, family *Peptococcaceae*, family *Defluviitaleaceae*, genus *Helicobacter*, genus *Ruminiclostridium_5*, genus *Defluviitaleaceae_UCG_011*, genus *Eubacterium__fissicatena_group*, genus *Ruminococcaceae_UCG_004*, and genus *Tyzzerella* were also increased in HFCurd mice. Furthermore, body weight was not significantly increased after 1 week of HF diet feeding [*F*_(__2_,_27__)_ = 0.285, *p* = 0.7542, Supplementary Figure 2C], although the accumulative energy intake was increased (F_(__2_,_27__)_ = 58.42, *p* < 0.0001, Supplementary Figure 2D]. Curdlan supplementation did not alter the energy intake and body weight of mice compared with HF group. These results indicate that acute curdlan supplementation prevented the microbial dysbiosis which appears in the early stage of HF diet treatment, before the onset of obesity and significant weight gain.

### Chronic Curdlan Supplementation Prevented HF Diet-Induced Cognitive Deficits

**FIGURE 2:**
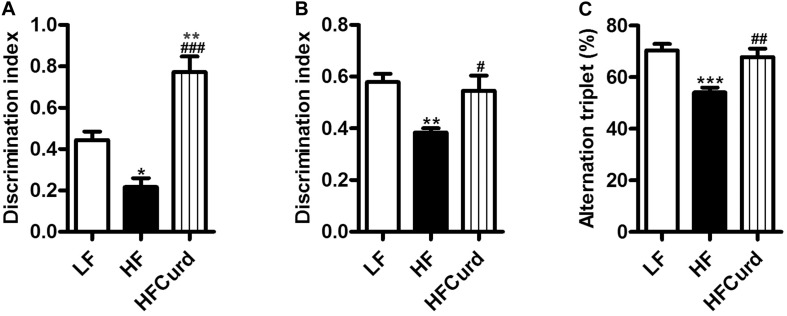
Effects of chronic curdlan supplementation on cognitive function in mice fed a HF diet. **(A)** Discrimination index in the temporal order memory test, **(B)** discrimination index in the novel object recognition test, and **(C)** proportion of correct alternations in the Y-maze test. Alteration triplet% was calculated as [number of successful alternations/(total number of arms entries −2) × 100]. Values are mean ± standard error of means. *n* = 10. *p < 0.05, **p < 0.01, ***p < 0.001 vs. LF; ^#^*p* < 0.05, ^##^*p* < 0.01, ^###^*p* < 0.001 vs. HF.

It has been reported that gut microbiota dysbiosis and obesity are strongly linked to cognitive decline (Zhang et al., 2019). Having established curdlan’s capacity to prevent gut dysbiosis, we next investigated if curdlan could prevent cognitive impairments induced by chronic HF diet. In the temporal order memory test, recognition memory was impaired in HF mice as evidenced by HF diet significantly decreasing the discrimination index compared to LF diet. Importantly, curdlan significantly increased discrimination index of HF mice, and the discrimination index of HFCurd mice was even higher than that of LF mice [*F*_(__2_,_27__)_ = 24.84, *p* < 0.0001, Figure 2A]. Similar results were observed in the novel object recognition test [*F*_(__2_,_27__)_ = 6.644, *p* = 0.0045, Figure 2B], further highlighting that recognition memory is improved with curdlan supplementation. In the Y-maze test, the proportion of spontaneous alteration in HF mice was markedly lower than that of the LF and HFCurd mice [*F*_(__2_,_27__)_ = 11.89, *p* = 0.0002, Figure 2C], suggestive of the deficits in spatial working memory caused by a HF diet, and these could be ameliorated by curdlan. These results indicate that cognitive deficits caused by a HF diet are preventable by chronic curdlan supplementation. Furthermore, we observed that the final body weight, plasma glucose, energy intake, liver mass, fat mass, and fat mass-to-body weight ratio were significantly higher in HF mice than LF mice (Table 1), while these abnormal metabolic parameters to some degree were improved by curdlan intervention (all *p* < 0.05, Table 1).

**TABLE 1:** The metabolic parameters in low-fat (LF) mice, high-fat (HF) mice and HF mice administered with curdlan for 15 weeks.

**Metabolic parameters**	**LF**	**HF**	**HFCurd**
Body weight (g)	28.2 ± 0.5	42.8 ± 2.2***	34.7 ± 1.3^##^
Plasma glucose (mmol/l)	5.1 ± 0.1	6.1 ± 0.3*	4.5 ± 0.4^##^
Energy intake (kcal/day)	11.4 ± 0.3	14.4 ± 0.3***	14.7 ± 0.3***
Plasma glucose AUC (mmol/l × min)	961.0 ± 31.5	1364.0 ± 51.6***	964.6 ± 36.2^###^
Liver mass (g)	1.4 ± 0.1	2.5 ± 0.3**	1.8 ± 0.1^#^
Liver mass/body weight (%)	4.6 ± 0.1	5.3 ± 0.4	4.2 ± 0.1^#^
Subcutaneous fat mass (g)	0.47 ± 0.04	1.9 ± 0.1**	1.1 ± 0.3^#^
Subcutaneous fat mass/body weight (%)	1.5 ± 0.1	3.7 ± 0.3***	2.1 ± 0.4^##^
Epididymal fat mass (g)	1.1 ± 0.2	2.7 ± 0.3***	1.69 ± 0.04^#^
Epididymal fat mass/body weight (%)	2.9 ± 0.4	5.9 ± 0.6***	4.3 ± 0.1^#^
Brown fat mass (g)	0.11 ± 0.01	0.27 ± 0.04**	0.14 ± 0.02^##^
Brown fat mass/body weight (%)	0.35 ± 0.01	0.54 ± 0.06*	0.30 ± 0.03^##^

Values are mean ± standard error of means. *n* = 10. *p < 0.05, **p < 0.01, ***p < 0.001 vs. LF; ^#^*p* < 0.05, ^##^*p* < 0.01, ^###^*p* < 0.001 vs. HF.

### Chronic Curdlan Supplementation Attenuated Hyperendotoxemia and Colonic Barrier Integrity Impairment and Inflammation Induced by HF Diet

**FIGURE 3:**
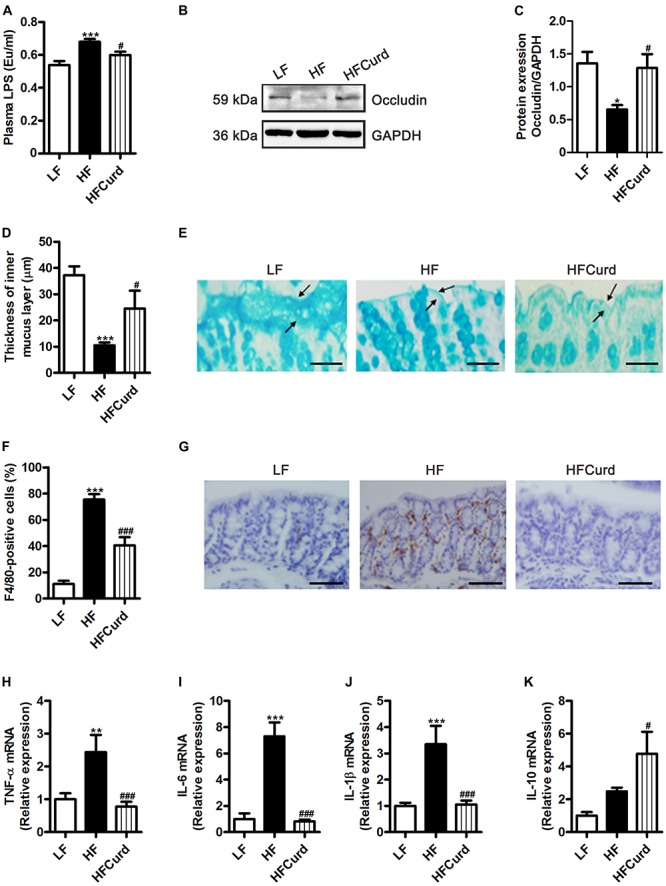
Effects of chronic curdlan supplementation on colonic barrier integrity and inflammation in mice fed a HF diet. **(A)** Plasma LPS levels, **(B,C)** protein expression levels of occludin in the colon, **(D)** quantification of colonic mucus layer thickness, **(E)** Alcian blue staining for colon. **(F,G)** Immunohistochemical staining and quantification of colonic F4/80-positive cells, and **(H–K)** mRNA expression of tumor necrosis factor-α (TNF-α) **(H)**, interleukin-6 (IL-6) **(I)**, interleukin-1βIL-1β **(J)**, and interleukin-10 (IL-10) **(K)** in the colon. Values are mean ± standard error of means. Values are mean ± standard error of means. *n* = 5. *p < 0.05, **p < 0.01, ***p < 0.001 vs. LF; ^#^*p* < 0.05, ^###^*p* < 0.001 vs. HF. Scale bar: 80 μM.

Gut microbiota dysbiosis is strongly linked to intestinal barrier damage and endotoxinemia (Cani et al., 2009). Firstly, we found that serum LPS levels were significantly increased with a chronic HF diet, which was reduced with curdlan supplementation, when compared to the HF group [*F*_(__2_,_12__)_ = 12.68, *p* = 0.0011, Figure 3A]. In addition to the hypoendotoxinemic effects, the colonic occludin expression was also decreased in mice fed on HF diet compared with the LF diet, while curdlan protected against the HF diet induced decline in colonic occludin expression [*F*_(__2_,_12__)_ = 5.565, *p* = 0.0195, Figures 3B,C], suggestive of an enhancement of the epithelial tight junctions. Using Alcian blue-stained sections to measure the colonic mucus layer, we found that HF diet significantly decreased colonic mucosal thickness in the colon of mice compared to LF diet, while curdlan increased mucosal thickness in the colon [*F*_(__2_,_27__)_ = 15.31, *p* < 0.0001, Figures 3D,E]. Furthermore, we examined colonic macrophage accumulation and inflammation in response to HF diet and curdlan supplementation. Compared with LF mice, the positive immunoreactivity of F4/80, the macrophage marker, was increased in the colon of HF mice, while curdlan supplementation prevented this increase [*F*_(__2_,_12__)_ = 41.43, *p* < 0.0001, Figures 3F,G]. In addition, curdlan administration-prevented HF diet increased mRNA level of pro-inflammatory cytokines tumor necrosis factor-α (TNF-α), interleukin-6 (IL-6), and interleukin-1β (IL-1β) in the colon (all *p* < 0.05, Figures 3H–J). Meanwhile, curdlan elevated the anti-inflammatory cytokine interleukin-6 (IL-10) levels, although there was no significant difference in the IL-10 expression between LF mice and HF mice [*F*_(__2_,_12__)_ = 12.1, *p* = 0.0013, Figure 3K]. Collectively, the results described above clearly indicate that curdlan supplementation prevented HF diet-induced hyperendotoxemia, colonic barrier integrity impairment, and colonic inflammation.

### Chronic Curdlan Supplementation Mitigated Microgliosis and Inflammation in the PFC and Hippocampus in Mice Fed HF Diet

**FIGURE 4:**
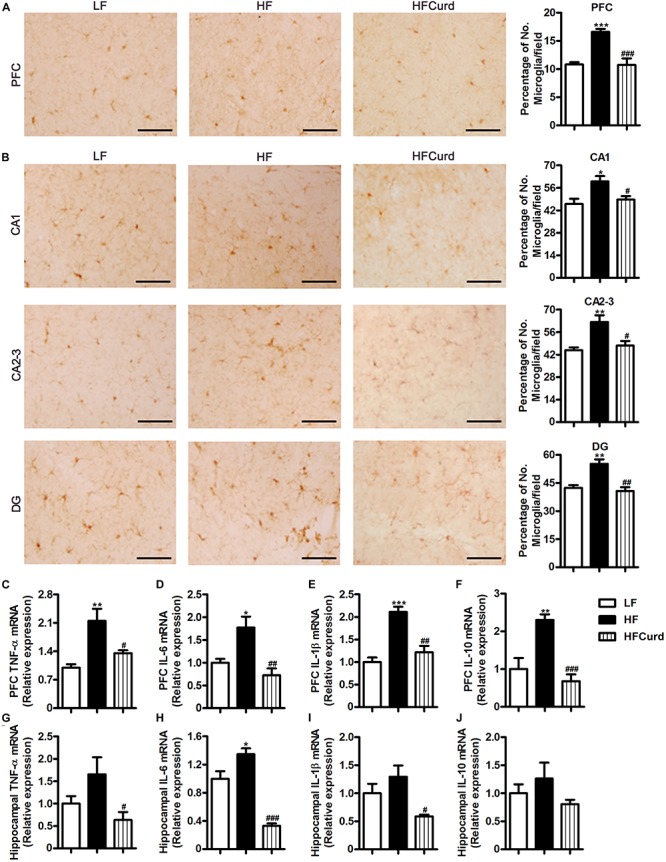
Effects of chronic curdlan supplementation on microgliosis and inflammation in the prefrontal cortex (PFC) and hippocampus of mice fed a HF diet. **(A,B)** Immunohistochemistry staining of Iba1 in the PFC **(A)** and the cornus ammonis (CA1), CA2-3, and dentate gyrus (DG) region of hippocampus **(B)**; **(C–J)** mRNA expression of TNF-α, IL-6, IL-1β, and IL-10 in the PFC **(C–F)** and hippocampus **(G–J)**. Values are mean ± standard error of means. *n* = 5. *p < 0.05, **p < 0.01, ***p < 0.001 vs. LF;^#^*p* < 0.05, ^##^*p* < 0.01,^###^*p* < 0.001 vs. HF. Scale bar: 40 μM.

Intestinal barrier impairment and endotoxinemia can mediate microglial activation and neuroinflammation (Cattaneo et al., 2017). After establishing the ability of curdlan to prevent colonic barrier damage and hyperendotoxemia, we then investigated the effects of curdlan on microgliosis and neuroinflammation induced by HF diet. Using Iba1 as an immunohistochemical marker of microglia, we verified that the HF diet was able to increase the number of microglia in the PFC and hippocampal regions, including cornus ammonis (CA1), CA2-3, and dentate gyrus (DG) region, while curdlan supplementation significantly reduced microglial numbers (all *p* < 0.05, Figures 4A,B). Furthermore, compared with LF group, HF group had a significant increase in the mRNA levels of TNF-α, IL-1β, and IL-6 in PFC region, and elevated IL-6 mRNA levels in hippocampal region. However, curdlan HFCurd mice exhibited the reduced mRNA levels of these genes (all *p* < 0.05, Figures 4C–E,G–I). In addition, curdlan supplementation-inhibited HF diet increased IL-10 mRNA expression in the PFC [*F*_(__2_,_12__)_ = 17.67, *p* = 0.0003, Figure 4F], but not in the hippocampus [*F*_(__2_,_12__)_ = 1.99, *p* = 0.1794, Figure 4J]. These results indicate that curdlan could prevent microglial activation and neuroinflammation in the PFC and hippocampus induced by HF diet.

### Chronic Curdlan Supplementation Ameliorated Synaptic Impairment in the PFC and Hippocampus of Mice Fed HF Diet

**FIGURE 5:**
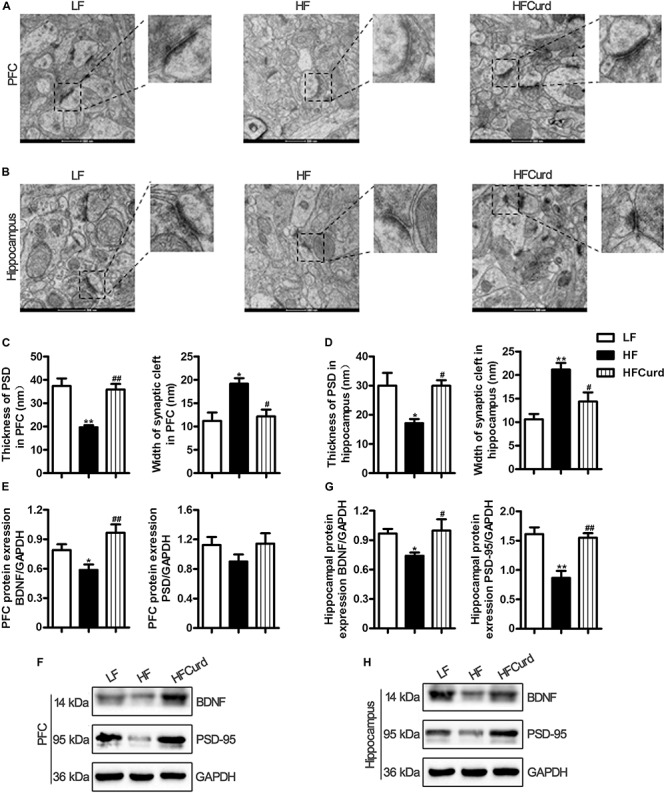
Effects of chronic curdlan supplementation on synaptic morphology and synaptic proteins in the PFC and hippocampus of mice fed a HF diet. **(A,B)** Electron micrograph of synaptic ultrastructure in the PFC **(A)** and hippocampus CA1 region **(B)**; **(C,D)** image analysis of thickness of postsynaptic densities (PSD), width of synaptic cleft and curvature of synaptic interface in the PFC **(C)**, and hippocampus **(D)**; **(E–G)** protein expression levels of brain derived neurotrophic factor (BDNF) and PSD-95 in the PFC **(E,F)** and hippocampus **(G,H)**. Values are mean ± standard error of means. *n* = 5. *p < 0.05, **p < 0.01 vs. LF; ^#^*p* < 0.05, ^##^*p* < 0.01 vs. HF. Scale bar: 500 nM.

Microglial activation and neuroinflammation are considered to be risk factors for cognitive decline and involved in the pathogenesis neurodegenerative diseases (Xiang et al., 2006; Subhramanyam et al., 2019). Synaptic ultrastructure and plasticity protein closely correlate with learning and memory functions (Bocarsly et al., 2015). We next sought to identify curdlan’s effects on the synaptic ultrastructure and quantify plasticity protein levels. Synaptic ultrastructure in the PFC and hippocampal CA1 region was specifically analyzed using TEM. We found that the HF diet decreased the thickness of the postsynaptic densities (PSD), broadened the synaptic cleft, and reduced the curvature of synaptic interface (all *p* < 0.05, Figures 5A–D). However, compared with HF diet, curdlan supplementation attenuated these synaptic ultrastructure alterations, showing thicker PSDs and a narrower synaptic cleft (all *p* < 0.05, Figures 5A–D). Meanwhile, we also measured the protein levels of synapse plasticity markers, BDNF, and postsynaptic density-95 (PSD-95) in the PFC and hippocampus of mice. In the PFC, BDNF expression, but not PSD-95, was inhibited by HF diet; however, this decrease in BDNF was not evident in the HFCurd mice [BDNF: *F*_(__2_,_12__)_ = 11.5, *p* = 0.0016, PSD-95: *F*_(__2_,_12__)_ = 0.9973, *p* = 0.3975, Figures 5E,F]. In the hippocampus, both BDNF and PSD-95 were decreased in HF mice; however, neither of these reductions were observed in HFCurd mice [BDNF: *F*_(__2_,_12__)_ = 5.698, *p* = 0.0182, PSD-95: *F*_(__2_,_12__)_ = 13.72, *p* = 0.0008, Figures 5G,H]. These results indicate that curdlan could improve synaptic morphology and plasticity in the PFC and hippocampus.

## Discussion

Although curdlan, an insoluble β-(1, 3)-glucan has been widely used as food additive, its potential effects on the gut-brain axis and cognition represent a gap in the current literature. In the current study, we first demonstrated that acute dietary supplementation with curdlan prevented the microbial dysbiosis induced by a HF diet, which preceded the significant body weight gain, characteristic of obesity. Importantly, chronic curdlan supplementation significantly improved cognition impairment in HF diet fed mice. In line with improved cognitive function, we found that chronic curdlan supplementation attenuated the neuropathology in the PFC and hippocampus, including inhibition of microgliosis, mitigation of neuroinflammation, and improvement of synaptic impairments. In addition, chronic curdlan supplementation significantly improved colonic barrier integrity, decreased serum LPS levels, and attenuated colonic inflammation. Overall, the results of acute and chronic experiment indicate that curdlan, as a prebiotic, mitigated HF diet-associated cognitive impairments, which is attributed to the improvement of colon-brain axis.

Previously, we have reported that gut microbiota dysbiosis plays an important role in the development of obesity-induced cognitive impairments by chronic HF diet (Wang et al., 2017; Zhang et al., 2019). Many studies including ours have concluded that obese mice fed chronic HF diet for 8–22 weeks showed the alteration of the richness, diversity, and composition of gut microbiota (Turnbaugh et al., 2008; Wang et al., 2017; Zhang et al., 2019), such as decreased Chao index and Shannon index, and increased representation of bacteria belonging to the Firmicutes phylum and a decrease in the Bacteroidetes. In contrast, relatively few studies have investigated the gut microbiota changes that occur prior to the onset of body weight gain in this model. In the present study, acute HF diet feeding for 7 days altered gut microbiota composition with a significant shift observed in the Firmicutes/Bacteroides ratio (Figure 1). This same trend has been noted previously in chronic diet-induced obesity and cognitively impaired mice (Wang et al., 2017; Zhang et al., 2019). Together, these findings demonstrate that significant, diet-related alterations of the gut microbiota composition upon short-term HF diet feeding were observed prior to the onset of obesity-induced cognitive impairment. Although the composition of gut microbiota was altered, the richness (Chao index) and diversity (Shannon index) were not significantly changed after acute HF diet feeding in the present study, indicating that the shift in the composition of gut microbiota occurs before any changes in microbiome diversity and richness during HF diet feeding. Importantly, we found that supplementation of dietary curdlan prevented this shift in microbiota composition, as it significantly increased Bacteroides and decreased Firmicutes (Figures 1A,B). In clinical studies, microbiota belonging to phylum Bacteroidetes has been shown to be related not only with obesity (Hu et al., 2015; Kasai et al., 2015) but also with cognition and neurodegenerative diseases (Carlson et al., 2018; Saji et al., 2019). For example, infants with high levels of gut Bacteroides at 1 year of age show higher cognitive ability at 2 years old (Carlson et al., 2018). In a cross-section study, a lower abundance of Bacteroides at genus is reported in the gut microbiota of dementia patients (Saji et al., 2019). At species level, Bacteroides fragilis was lower in patients with cognitive impairment and brain amyloidosis (Cattaneo et al., 2017). Here, we found that not only abundance of Bacteroidetes phylum was increased with curdlan supplementation, but the family Prevotellaceae, genus Alloprevotella, family Rikenellaceae, and genus Alistipes (all belonging to Bacteroidetes phylum) were also increased (Figures 1A,F). These results suggest that curdlan could be administered as a prebiotic to enhance the abundance of certain members of bacterial community belonging to Bacteroidetes phylum, which contribute to improve cognition in obesity.

It is noteworthy that the gut microbiota is an important regulator of host intestinal barrier integrity and endotoxemia (Desai et al., 2016). Consistent with previous studies (Cani et al., 2007; de La Serre et al., 2010; Slyepchenko et al., 2016), we found that a HF diet dramatically increased intestinal inflammation and diminished intestinal barrier integrity, which may result in the release of bacterial LPS into the bloodstream (Figure 3). Importantly, we found that chronic curdlan supplementation enhanced colonic mucus thickness and increased colonic tight-junction protein levels, indicating that curdlan could prevent the loss of intestinal barrier integrity seen with a HF diet (Figure 3). Although the exact reason is not clear, it is known that an outer membrane protein of Bacteroidetes can bind to polysaccharide (Tuson et al., 2018). Bacteroidetes genomes encode many polysaccharide lyases and glycoside hydrolases, largely involved in the acquisition and metabolism of polysaccharides (Thomas et al., 2011). Therefore, curdlan as polysaccharide may favor the development of the polysaccharide-degrading Bacteroidetes and its next taxonomic levels observed in the present study. It has been reported that Bacteroidetes alone or in conjunction with other gut microbiota, benefit their host mucus and gut barrier (Bäckhed et al., 2005). For example, Bacteroides fragilis decreases gut permeability in the maternal immune activation mouse model that is known to display features of autism spectrum disorder (Hsiao et al., 2013). Co-colonization of Bacteroides thetaiotaomicron with Eubacterium rectale, the most common gut microbiota, increases the expression of genes to direct the synthesis of mucosal glycans, including α-1,2 fucosyltransferase, α-1,3-fucosyltransferase, glycosphingolipids, and *O*-glycans (Mahowald et al., 2009). Therefore, curdlans serve as platform elements which can be fermented by Bacteroidetes, to provide an energy source for bacteria within the Bacteroidetes phylum and other gut microbiota. Resultantly, the production of mucosal glycans were evidently increased and this enhancement of the mucus layer offered greater protection against the epithelial damage induced by HF diet. Indeed, we found that the expression of the intestinal tight junction protein, occludin, was increased in the curdlan supplemented group (Figures 3B,C), which may also contribute to the reduction of gut permeability and translocation of bacterial LPS into the blood circulation.

It is reported that LPS from the intestinal tract was increased in the cortex and hippocampus of AD patients (Cattaneo et al., 2017). Notably, the presence of bacterial components has been observed in the post-mortem brain tissue of AD patients (Emery et al., 2017), which indicates that the increased gut permeability and hyperndotoxinemia could contribute to AD pathology. Our data illustrated that curdlan enhanced the intestinal barrier and resulted in a profound reduction in endotoxinemia, which may contribute to the improvements in cognition we observed by a comprehensive array of behavioral, learning, and memory tests in the present study. Neuroinflammation is considered to be the link between gut dysbiosis to synaptic and cognitive decline, while it is also one of key mechanisms underlying various neurodegenerative diseases (Giau et al., 2018). Overexposure to LPS by intraperitoneal injection induced microglial activation and increased expression of pro-inflammatory cytokines in the brains of mice (Chen et al., 2018). Here, we found that a chronic HF diet increased microglial accumulation and pro-inflammatory cytokine expression in the PFC and hippocampus which were both attenuated by curdlan supplementation, suggesting curdlan has an antineuroinflammative effect (Figure 4). A growing body of evidence demonstrates the crucial role of microglia in mediating the cognitive dysfunction observed in neurodegenerative disorders, for example in the AD brain where an overpruning of synapses has been observed (Hong et al., 2016). Synaptic structure and plasticity are closely correlated with learning and memory functions (Bocarsly et al., 2015). Dysregulation of synaptic formation and plasticity in the hippocampus have been implicated in the patients with cognitive impairment and AD (Head et al., 2009; Whitfield et al., 2014). Here, we found that prolonged HF diet for 15 weeks damaged ultrastructural synaptic architecture in the PFC and hippocampus characterized by decreased PSD thickness and broadened synaptic cleft with TEM technique. Importantly, chronic curdlan supplementation prevented the HF diet-induced damage to the ultrastructural synaptic architecture (Figures 5A–D). In line with these findings, we also found that curdlan reversed HF diet-associated decreases in the molecular markers of synaptic plasticity, BDNF, and PSD-95 in the PFC and hippocampus (Figures 5E–H). Therefore, curdlan supplementation evidently improved ultrastructure and increased synaptic protein expression which supports the enhancement and maintenance in cognitive function despite chronic HF diet feeding.

In a previous study of the diet-induced obese mouse model, the M1 pro-inflammatory phenotype macrophage was activated in the colon as evidenced by increased pro-inflammatory cytokine TNF-α, IL-6, and IL-1β expression, as well as CD11c positive staining macrophage in the colon. However, the M2 anti-inflammation phenotype was not significantly altered in obese model without alterations in CD206 positive staining (Zhang et al., 2019). Therefore, it suggests that in obesity, the M1 pro-inflammatory macrophage was activated in colon, while the M2 phenotype, the most normally resident macrophages in the colon, was less affected. Therefore, in the current study, we examined three markers of pro-inflammation TNF-α, IL-6, and IL-1β to investigate curdlan’s effect on inhibition of M1 activation and inflammation in the gut and brain. It has been reported that LPS promoted inflammation with activation of M1 macrophages (Zheng et al., 2013). Therefore, the hyperendotoxemia in obese mice may contribute to the activation of M1 pro-inflammatory macrophage or microglia in the colon and brain (Figures 3H–J, 4C–E,G–I). Importantly, we found that the curdlan has shown a great ability to inhibit the activation of M1 pro-inflammatory cytokines, TNF-α, IL-6, and IL-1β, while IL-10 is secreted from M2 macrophage activation (Xu et al., 2019). In the present study, we found that the level of IL-10 in gut and brain is ambiguous in obese model and curdlan intervention. IL-10 expression and M2 phenotype polarization in macrophage are regulated by mitochondria repurposing (Mills et al., 2016). For example, inhibition of succinate dehydrogenase (SDH), mitochondrial hyperpolarization, and reactive oxygen species (ROS) production will drive M2 polarization and increase IL-10 level in macrophage (Mills et al., 2016). In future studies, other markers of M2 macrophage and metabolic repurposing of mitochondria should be investigated to further examine the anti-inflammation polarization status of macrophage or microglia with curdlan intervention cognitive impairment mice induced by obesity.

Research showed that humans continue to consume less fiber than the recommended 25–35 g/day by the World Health Organization (Lattimer and Haub, 2010). The diets of Americans, Australians, and Chinese have experienced a decrease in fiber intake (Clemens et al., 2012; Wang et al., 2014; Yu and Lu, 2012). Dietary fiber intake is closely related with cognitive function (Kentner et al., 2016; Makki et al., 2018). Cross-section studies found that dietary fiber intake is positively correlated with improved cognition in pre-pubertal individuals in the US and elderly people in Korea (Lee et al., 2001; Khan et al., 2015). Curdlan is an insoluble polysaccharide, which has been considered as dietary fiber and additive in food. We found that chronic curdlan administration improved recognition and spatial working memory in the temporal order memory, novel object recognition, and Y-maze test in HF mice (Figure 2). Furthermore, the discrimination index of HFCurd mice was even higher than that of LF mice in the temporal order memory test (Figure 2A). Although, during control diet feeding, there was no statistically significant difference in cognition index of behavior tests between curdlan supplementation (LFCurd) group and LF group (in Supplementary Figure 3), the index of LFCurd mice was higher than LF mice in statistical trend (*p* = 0.0632) in the temporal order memory test. Overall, these results suggest that curdlan is mainly able to counteract HF diet negative effects in cognition, and to some degree to improve temporal order memory in absent of HF diet. Therefore, dietary curdlan intake may have a potential to prevent cognitive impairment induced by overconsumption of the Western diet.

In this study, the effects of curdlan on cognition improvement was only examined in male obese mice. The study of male mice was performed because it is reported that male mice develop a greater extent of cognition deficits induced by obesity or diabetes than female mice (Fan et al., 2018). Furthermore, the study of male mice may avoid the effects of estrogen and ovaries on cognition behavior. Moreover, studies have shown sex differences in obesity and neuroinflammation (Fan et al., 2018; Link and Reue, 2017). For example, it has been reported that females are protected from the Western diet-induced inflammatory response due to the protective effects of estrogen receptor α (Sample and Davidson, 2018), while male mice have greater inflammation in the central nervous system in multiple sclerosis animal model (Du et al., 2014). Therefore, the effects of curdlan supplementation on the cognition and gut-brain axis in female mice should be further examined in future study prior to the translation of curdlan into female human clinical trials.

In summary, the present study has demonstrated that dietary curdlan supplementation prevents cognitive deficits induced by a HF diet in mice. These beneficial effects can be attributed to the colon-brain axis. Our findings provide evidence of the mechanism by which curdlan—as food additive and prebiotic—can be used in the prevention of cognitive impairment induced by overconsumption of the Western diet.

## Data Availability Statement

All datasets generated for this study are included in the article/Supplementary Material.

## Ethics Statement

The animal study was reviewed and approved by The ethics committee of Xuzhou Medical University.

## Author Contributions

XY, WP, PZ, MH, X-FH, and YY designed the research study. MZ, HS, and DL performed the research. XY, MZ, HS, XC, PZ, and YY analyzed the data. XY and YY wrote the manuscript. KZ and X-FH reviewed the manuscript. All authors approved the manuscript.

## Conflict of Interest

The authors declare that the research was conducted in the absence of any commercial or financial relationships that could be construed as a potential conflict of interest.
