# Supplementary material for: Curdlan Prevents the Cognitive Deficits Induced by a High-Fat Diet in Mice via the Gut-Brain Axis
Source: Front Neurosci. 2020 May 14;14:384. doi: 10.3389/fnins.2020.00384 (PMC7239995; doi:10.3389/fnins.2020.00384)
Supplement: Supplementary file 1 [file Data_Sheet_1.pdf]

# *Supplementary Material*

## Supplementary Figures

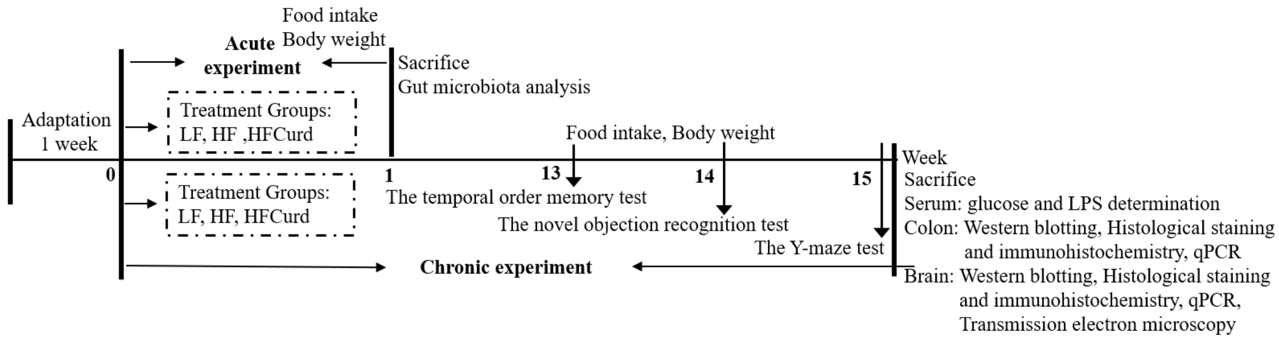


**Supplementary Figure 1.** Schematic figure of the experimental timeline.

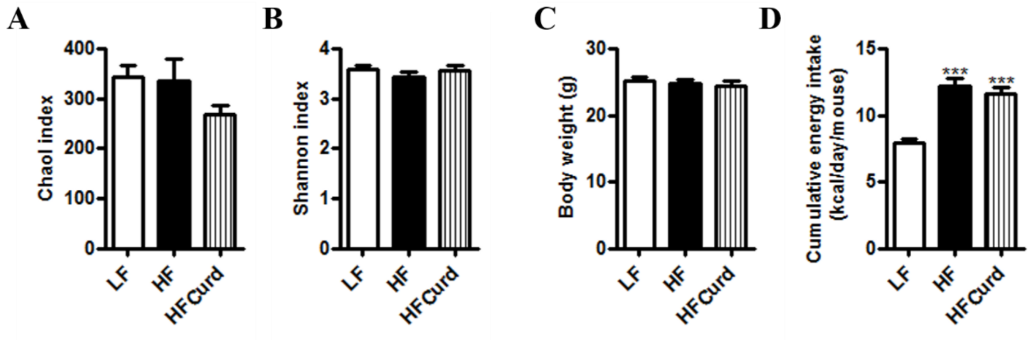


**Supplementary Figure 2.** Effect of curdlan on species richness and diversity, energy intake and body weight in mice fed a HF diet for seven days. (A) richness (Chao1 index) and (B) diversity (Shannon index) of gut microbial communities based on OUT, n=5; (C) body weight, (D) energy intake, n=10. Values are mean ± standard error of means. ***p<0.001 vs. LF.

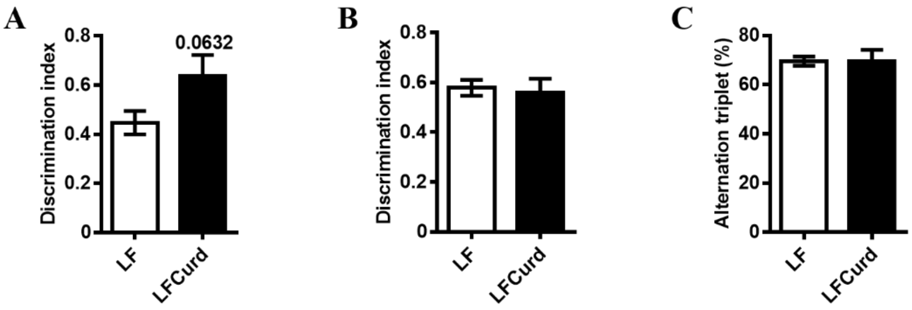


**Supplementary Figure 3.** Effects of chronic curdlan supplementation on cognitive function in mice fed a LF diet. (A) discrimination index in the temporal order memory test, (B) discrimination index in the novel object recognition test, (C) proportion of correct alternations in the Y-maze test. Alteration triplet %: was calculated as [number of successful alternations/(total number of arms entries-2) × 100]. n=10. Values are mean ± standard error of means.

**Supplementary Tables**

**Supplementary Table 1. Primers used for qPCR**

| Mouse Gene | Forward Primer (5′→3′) | Reverse Primer (5′→3′) |
|---|---|---|
| TNF-$\alpha$ | CTTGTTGCCTCCTCTTTTGCTTA | CTTTATTTCTCTCAATGACCCGTAG |
| IL-1β | TGGGAAACAACAGTGGTCAGG | CTGCTCATTCACGAAAAGGGA |
| IL-6 | TCACAGAAGGAGTGGCTAAGGACC | ACGCACTAGGTTTGCCGAGTAGAT |
| IL-10 | GGAAGACAATAACTGCACCCACT | CAACCCAAGTAACCCTTAAAGTCC |
| β-actin | TGAGAGGGAAATCGTGCGTGAC | GCTCGTTGCCAATAGTGATGACC |
